# Lessons from a genome-wide CRISPR-Cas9 screening: what researchers should know before start

**DOI:** 10.17179/excli2021-4412

**Published:** 2021-12-06

**Authors:** Mohammad Masoudi

**Affiliations:** 1 Department of Biological Sciences, Institute for Advanced Studies in Basic Sciences (IASBS), Zanjan 45137-66731, Iran

## ⁯⁯

***Dear Editor,***

The CRISPR technology has revolutionized the biomedicine field in a way like of which has rarely been seen before and PCR might be the only example one can think of. This system, originally found in bacteria as an adaptive immune system (Makarova et al., 2011[2]), soon proved to be one of the most powerful tools in biomedicine with its application in gene manipulation including knockout, suppression, activation, editing (Adli, 2018[1]), functional studies and therapeutics (Steinhart et al., 2017[6]; Uddin et al., 2020[7]). Large-scale screening is a CRISPR application been used for finding genes involved in a biological pathway of interest. With its ability to target and knockout any gene of interest, CRISPR-Cas9 system has been used in a variety of genome-wide screening studies where a library of sgRNAs is screened for the spacers whose targets are involved in a process of interest (Shalem et al., 2014[5]; Wang et al., 2014[8]). These functional genomic studies have led to introduction of new potential therapeutic targets that had not been considered before the CRISPR era. An example of these studies is the genome-wide CRISPR-Cas9 screening that we performed and led to the introduction of *SH3D21* as a novel sensitizer for gemcitabine (Masoudi et al., 2019[3]). Given the complex and multistep nature of a CRISPR-Cas9 genome-scale screening (see supplementary information, Supplementary Figure 1), there are some critical points and challenges that a researcher should be aware of before start. In this review, the experience of a genome-wide CRISPR-Cas9 screening is shared with researchers who are considering to use this system for their large-scale screening and don't have an experience of doing so. The author walks the readers through the process step-by-step, mentions his experience/challenges and wherever applicable shows original results as examples. 

### Choosing the topic and the cell line

Performing a genome-scale CRISPR-Cas9 screening is a multistep process, the very first step of which is choosing the right topic and the cell line (see supplementary information, Supplementary Figure 1). It is important to choose a topic that has the capacity for a large-scale screening. Some topics seem appealing however in reality they don't have enough capacity for a genome-wide screening. The numbers should be kept in mind when considering performing these kinds of large-scale projects. The entire project should be written down on paper and the numbers and procedures should be thought of carefully. For example, GeCKO v2 sgRNA libray has 123,411 sgRNAs (Sanjana et al., 2014[4]). One should know what his/her starting number of the cells would be (how many time of the library) and what number he/she would expect to collect after the treatment. If the surviving cells are in a limited number, the researcher will not be able to collect the majority of the sgRNA library after treatment; and that would make it difficult to have reliable result out of the screening.

It is also important to pick the right cell line. Some cell lines may be too sensitive to lentiviral infection or antibiotic treatment (usually used for the selection step). So, some preliminary experiments, in smaller scale, are needed to test if the cell line of choice is suitable for such an experiment. 

### Choosing the sgRNA library

If researchers don't have the intention to design, make and use their own sgRNA library, they can choose from a list of sgRNA libraries from different labs available for use in different organisms (https://www.addgene.org/crispr/libraries/). In selecting the sgRNA library there are some important points that should be considered: 1) Single or double vector library. For the CRISPR-Cas9 system to work the sgRNA and the Cas9 protein need to be expressed within a cell simultaneously. Some libraries use double vector system, meaning the Cas9 and sgRNA expressing sequences are on two different vectors. Therefore, at first researchers need to prepare Cas9 expressing cells from their cell line of interest, then transduce those cells with the sgRNA library. On the other hand, there are some libraries that use single vector system, meaning Cas9 and sgRNA expressing sequences are located on a single vector. In that case, one single transduction is enough to express the Cas9 and sgRNA in a cell at the same time and have the gene of interest knocked out. Since researchers don't have to perform sequential transduction, the single vector system is easier and cheaper to work with unless they have some special considerations. 2) Number of sgRNAs per gene in the library. It is important to have enough number of sgRNAs per gene in the library of choice since not all the sgRNAs work with the similar efficacy. In general, the more the sgRNA per gene, the better the results. 3) Have the other groups besides the original developer group used the library? Application of the library by different groups is an important point that shows how reliable and easy-to-use a library is. 

### Preparing the plasmid library

After purchasing/obtaining the sgRNA library of choice it should be expanded at home, which requires transformation of a host bacterium with the library and extracting the expanded plasmids. An extensive care should be taken at this step since this prepared library will be the one that will be used for the project/s in hand or some other future projects. When the plasmid extraction is done, the author highly recommends the researchers to check the coverage and dynamic range of the sgRNA library by sequencing, to make sure that the prepared library covers all the sgRNAs and has enough copy number of each sgRNA in it.

### Packaging the plasmids into virus particles

Once the plasmid library is prepared, it needs to be packaged into virus particles. It is better to start with a small scale and perform some preliminary experiments to reach the best protocol for transfecting the cells and colleting the virus particles. After collecting the virus particles from the medium, it is beneficial to have them as concentrated as possible since it is easier to work with and gives researchers more choices in the next step. The virus particles can be concentrated with a single centrifugation step. 

### Transduction with virus particles and selection with antibiotics

This step is a crucial step since the integrity of the screening is on the line. Once the plasmids are packaged into virus particles, it is time to transduce the cells with the virus particles. Exhaustive care must be taken to infect the cells in a way that they receive only one virus particle (containing a single sgRNA). To achieve this goal the cells are usually infected with the multiplicity of infection (MOI) ≤ 0.3. Meaning only one cell out of three will be transduced with a virus particle. Preliminary experiments are needed to obtain the amount of the virus that is needed to reach this MOI. 

After the transduction, the antibiotic selection step is reached. One important point in this step is the concentration of the selection antibiotic. Some pilot experiments must be performed to discover the best concentration of the antibiotic for the cells in hand. If the concentration of the antibiotic is too high, some of the transducted cells will be lost during antibiotic selection. And if the concentration is too low, there will be some untransducted cells left after the selection. Usually, the minimum dose of the antibiotic that kills the cells within 3 days should be used. The selection time for the screening must be chosen in a way to make sure that enough time has been given to the antibiotic to work. 

### Screening

Following the selection of the cells with the antibiotic, the result will be a library of the cells each carrying an sgRNA. If researchers wish to perform a gene essentiality screening, the only task they need to do in this step is to cultivate the library for enough time to have cells carrying sgRNAs targeting essential genes eliminated from their library. There are some points that should be considered in this step. 1) The cells should be collected and freezed at different time points. In general, no extra cells from passaging should be thrown out. They should just be counted, labeled and freezed since they may be used in future analysis. 2) The size of the library should be chosen at the most affordable scale to collect all the spacers after the selection and the screening. This completely depends on the available budget, but the bigger the scale of the library the better the coverage and the better the result. 

### Treatment 

Some researchers are interested in essential genes for the survival of their cell line of interest. In that case they don't need to treat their prepared cell library and they merely cultivate the sgRNA carrying cell library for enough time to have cells with sgRNAs targeting essential genes out of the library. However, in some projects, like ours, one may wish to treat the cells after the primary selection and have a special screening for a pathway of interest. In that case, some points should be kept in mind while planning the project. 1) Selecting the cells with the antibiotic for a long enough time to make sure that the background genes have dropped out from the library. In any CRISPR-Cas9 screening, there are some cells carrying sgRNAs against the essential genes like the ones involved in protein synthesis or cell division. Normally, the number of these cells will drop after several doubling section of the cells. If not enough time is given to the library to eliminate these essential genes, they may appear as the top hits of the screening as false positives. 2) Choosing the timeline of the treatment carefully. Preliminary experiments are required to choose the best timetable for performing the treatment. 3) Making the scale big enough so that the entire sgRNA library is covered. 

### Next generation sequencing

When the treatment is complete, a next generation sequencing library needs to be prepared from the samples. For that genomic DNA of the samples will be extracted and the sgRNA segments that are now incorporated in the cells' genome will be amplified. Based on the sequencing platform used, different kind of sequencing primers may need to be attached to the amplicons. When the sequencing library is prepared and quantified, it will be subjected to sequencing by desired platform. One point to have in mind in this step; the sequencing scale should be as big as researchers can afford to have the entire library covered with an acceptable average read count.

### Data analysis

Once the sequencing step is over, there will be raw sequencing data in hand that needs to be processed to give the sgRNAs/spacers read count. Usually, this step is done by the bioinformatitions, but the author encourages the researchers to learn the computational biology skills and perform this step themselves, since they are the ones that have planned and performed the experiments and they know the nature of the data the best. As there are many details that are important when one is analyzing a sequencing data, the best way is that the same person who has produced the data analyzes it. In case it is not possible for a researcher to perform the data processing himself/herself, he/she should be in close contact with the bioinformatition to make sure that the data is analyzed as it should. 

### Read counts in hand

Done with raw sequencing data processing, the result will be the read counts of the sgRNAs in each of the samples. Now the followings should be done: 1) Checking the coverage and dynamic range of the data to make sure that the majority of the sgRANs have been collected successfully with an acceptable read count. 2) Making sure that the genome-scale knockout experiment has been successful. To do so, researchers can (a) Check the depletion of sgRNAs by comparing a sample that has been collected late with the one that has been collected early after antibiotic selection. For example, in our study we compared the sgRNAs read counts from samples of day 7 and day 22 (Masoudi et al., 2019[3]). (b) One also can use Gene Set Enrichment Analysis algorithm to make sure of the integrity of the genome-scale knockout experiment. Doing so, some essential gene sets, like protein synthesis machinery, should be depleted in the late-collected sample. 

In some projects, researchers wish to treat the knockout cell library and screen the sgRNAs for a specific trait. For example, in our study we used the sgRNA carrying cell library of Panc1 cells to screen for the genes involved in gemcitabine modulation. In that case, the query of the genes with differential effect between the treated and un-treated samples is of interest. An algorithm that is widely used for analysis of such data is RNA Interference Gene Enrichment Ranking (RIGER). RIGER ranks the genes based on the rank of their targeting elements, siRNAs/shRNAs or sgRNAs (in case of CRISPR-Cas9 screenings). Since for knockout of each gene a number of sgRNAs are used, e.g. 6 sgRNAs per gene in GeCKO v2 library, and each sgRNA has its own efficacy, the differential effect of the gene should be calculated seeing these different efficacies all together. One method of RIGER algorithm, weighted sum method, ranks the genes based on the rank of their top two sgRNAs. This is particularly beneficial since not all the sgRNAs used in a library have the same efficacy or have been validated by the developer laboratory. 

### Validation experiments

Having the genes ranked based on their differential effects, now the genes with most influence on the trait of interest has appeared as top hits of the ranked list. One important point to have in mind is not to take these results as guaranteed. The top hits of the list must be validated by follow-up experiments to make sure that the result coming out of the large-scale screening for an individual gene is in fact reliable. To do so, one needs to do the following. (1) Preparing the cells that carry an sgRNA targeting the gene of interest. The sgRNA used should be the one that has been seen as the most effective in the screening. After transduction of the cells with the virus particles carrying the sgRNA and following antibiotic selection, to check if the sgRNA has correctly targeted the gene of interest researchers have some options. Taking advantage of western blotting to see if the level of protein has decreased in sgRNA carrying cells is one option. However, this can be tricky since a sharp decrease in amount of the protein of interest may not be seen as expected (see supplementary information, Supplementary Figure 2). The reason is that when the area of interest on the genome is targeted by Cas9 nuclease it produces double strand breaks at the site, which are then repaired by None Homologous End Joining (NHEJ) repair system resulting in mutations. Some of the mutations are protein-stabilizing mutations, which may be the reason why a dramatic decrease in amount of the protein is not seen after its coding gene has been targeted with an sgRNA. Therefore the better option to check whether the sgRNA has worked successfully on its target site is to check the mutations on the genome directly. For that, a SURVEYOR assay can be performed, which enables researchers to know whether they have indel (insertion or deletion) mutations on their target site. (2) The prepared sgRNA carrying cells are polyclonal and the effect that has been seen is the cumulative effect of all the clones. To have a more clear result, it is better to prepare monoclonal cell populations from the polyclonal cell population and perform the validation experiments using those clones as well. However, this may not be always possible since in some cases loss of function clones may be too delicate to tolerate harsh condition of monoclonal cell line preparation. In that case, the effect that has been seen in knockout cells should be confirmed with another method like siRNA/shRNA. One other way to have a better confidence about the result is to perform re-expression (rescue) experiment to see if the effect of sgRNA can be reversed by reintroducing the protein to the cell. (3) Last but not the least, researchers must be careful about the false positives. Some differential effects by sgRNAs of a certain gene may be seen in the genome-scale knockout screening, polyclonal cell population and even monoclonal cells, however, the effect seen may be a false positive phenomenon resulting from operation of the sgRNA on some other parts of the genome, which is called off-target activity. Extreme caution should be taken about this off-target operation of sgRNAs as it may not be detected unless by performing exhaustive validation experiments. In the following, an example of how the off-target activity of sgRNAs can be misleading is mentioned. 

In our genome-scale screening for gemcitabine modulators, *DNAJB12* gene appeared as one of the top sensitizers (Masoudi et al., 2019[3]) with two sgRNAs ranked 175 and 176 in top 1000 depleted sgRNAs list (see supplementary information, Supplementary Figure 3a). In validation experiments, we prepared cells carrying the 175-ranked *DNAJB12* targeting sgRNA. We performed gemcitabine dose-response experiment using the cells and observed that those cells were more sensitive to gemcitabine than cells carrying control sgRNAs (see supplementary information, Supplementary Figure 3b). We checked the target site of the sgRNA on *DNAJB12* gene and made sure that the target site was actually affected by the sgRNA (see supplementary information, Supplementary Figure 3c). To completely rule out the chance of off-target activity of the sgRNA, we prepared 14 cell lines from the polyclonal cells. We used those cell lines in cell viability experiments for gemcitabine and as expected observed that some of the cell lines were more sensitive to gemcitabine while others showed no difference with control cells or were less sensitive (see supplementary information, Supplementary Figure 3d). Then we checked the target site of the sgRNA on *DNAJB12* gene by Sanger DNA sequencing for individual cell lines and surprisingly none of the gemcitabine sensitive clones had their *DNAJB12* gene mutated at the sgRNA target site (see supplementary information, Supplementary Figure 4). This observation made it clear that the sensitizing effect of the *DNAJB12* targeting sgRNA was due to its off-target activity, a fact that could not be observed unless by the exhaustive validation experiments. 

### Conclusion

In this review, the author walked the readers through the process of performing a genome-scale knockout screening using CRISPR-Cas9 technology. It was tried to mention all the important points of which a researcher should be aware of before start of his/her screening and the points were accompanied with original data from a screening wherever applicable. Since this kind of genome-scale studies consume a lot of resources, the author recommends the researchers to plan their screening carefully beforehand and see all the steps through before start. Also, as it was made clear with an example, one needs to be extremely careful about reporting an individual gene as effective on his/her trait of interest. Comprehensive follow-up experiments should be performed to make sure the results coming out of a genome-scale experiment about a certain individual gene is actually valid. 

## Conflict of interest

The author declares that he has no conflict of interest.

## Supplementary Material

Supplementary information
