# Supplementary material for: Lessons from a genome-wide CRISPR-Cas9 screening: what researchers should know before start
Source: EXCLI J. 2021 Dec 6;20:1615–20. doi: 10.17179/excli2021-4412 (PMC8743829; doi:10.17179/excli2021-4412)
Supplement: Supplementary information [file EXCLI-20-1615-s-001.pdf]

EXCLI Journal 2021;20:1615-1620 – ISSN 1611-2156
Received: October 12, 2021, accepted: December 01, 2021, published: December 06, 2021

Supplementary information to:

Letter to the editor:

LESSONS FROM A GENOME-WIDE CRISPR-CAS9 SCREENING: WHAT RESEARCHERS SHOULD KNOW BEFORE START

Mohammad Masoudi

Department of Biological Sciences, Institute for Advanced Studies in Basic Sciences (IASBS), Zanjan 45137-66731, Iran. Tel: +98-24-33153313; E-mail: masoudi@iasbs.ac.ir

https://dx.doi.org/10.17179/excli2021-4412

This is an Open Access article distributed under the terms of the Creative Commons Attribution License (http://creativecommons.org/licenses/by/4.0/).

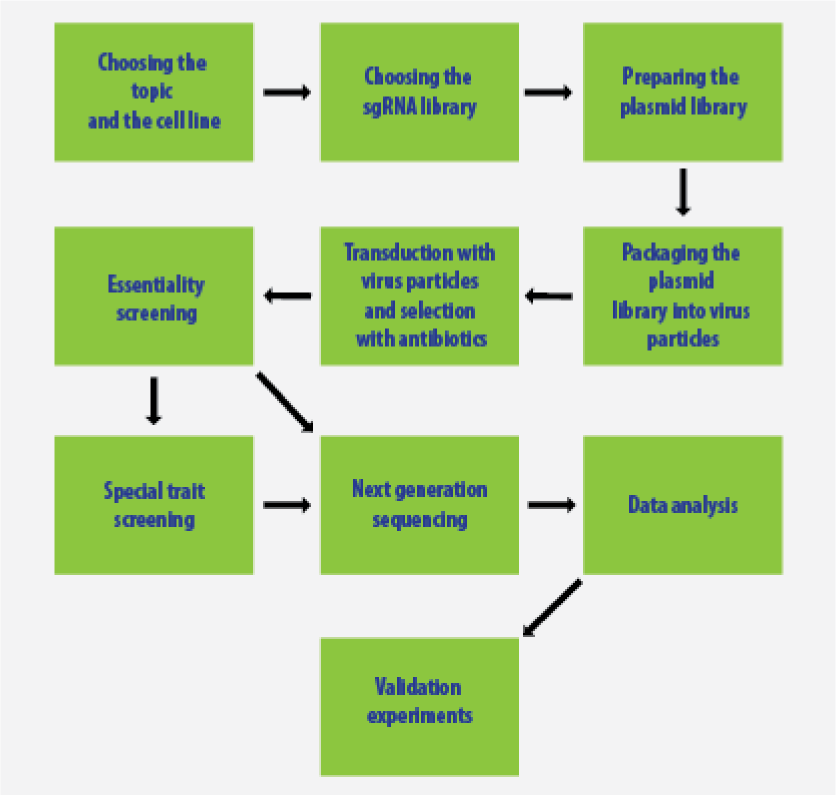

Supplementary Figure 1: Overview of a genome-scale CRISPR-Cas9 screening

EXCLI Journal 2021;20:1615-1620 – ISSN 1611-2156
Received: October 12, 2021, accepted: December 01, 2021, published: December 06, 2021

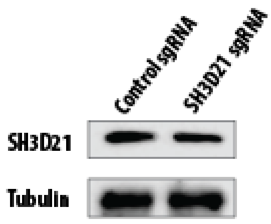

Supplementary Figure 2: Western blotting of SH3D21 protein in *SH3D21*-knockout cells. The amount of SH3D21 protein was assessed in *SH3D21*-knockout cells. As seen here the quantity of SH3D21 protein didn’t show a dramatic decrease in knockout cells while the mutations on the *SH3D21* gene was confirmed by SURVEYOR assay (Masoudi et al., 2019).

EXCLI Journal 2021;20:1615-1620 – ISSN 1611-2156
Received: October 12, 2021, accepted: December 01, 2021, published: December 06, 2021

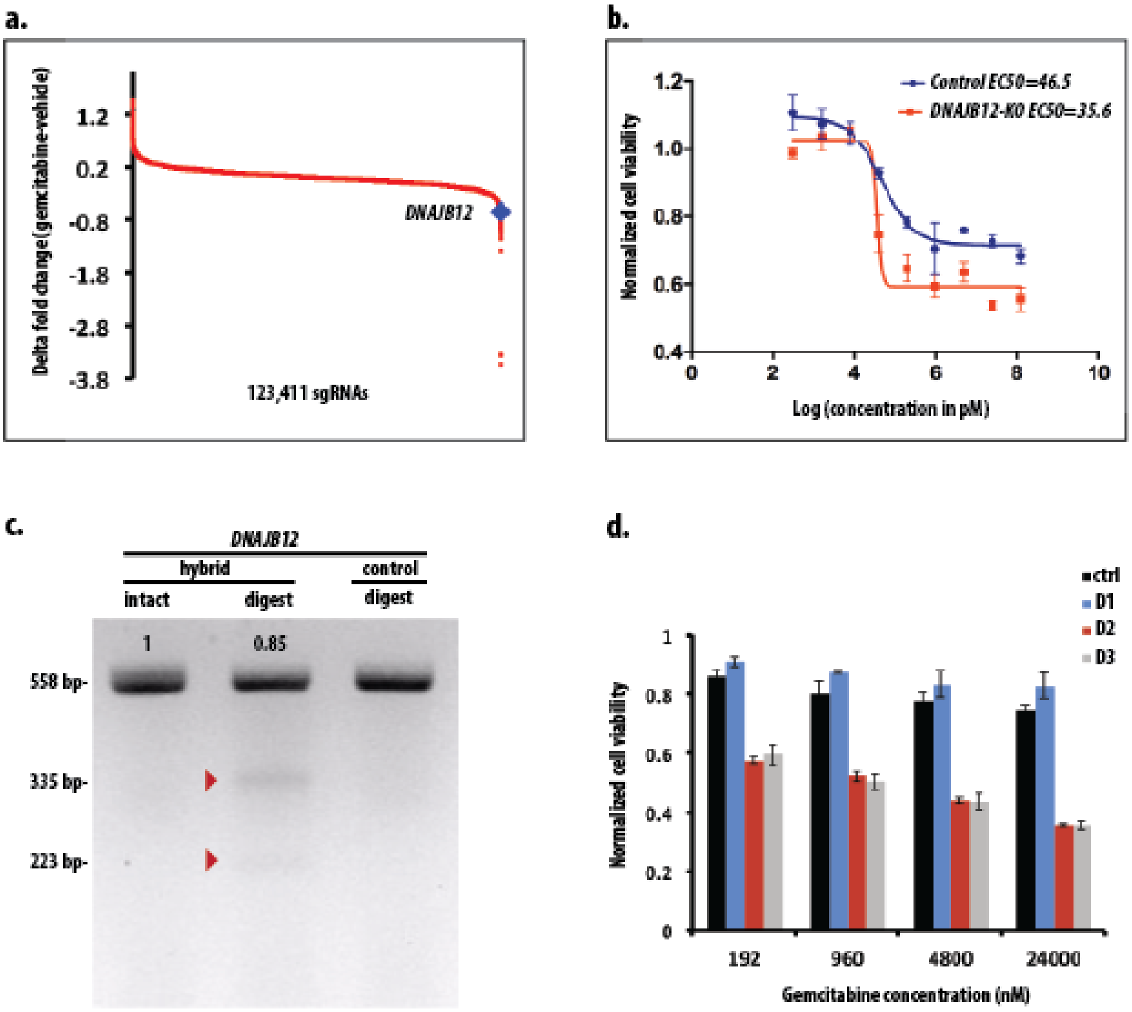

Supplementary Figure 3: *DNAJB12* as a gemcitabine sensitizer candidate. The gene *DNAJB12* appeared as one of the top hits in gemcitabine sensitizers list (Masoudi et al., 2019). **a**) Position of top two *DNAJB12* sgRNAs amoung the 123, 411 sgRNA of the library after treating the Panc1 cells with gemcitabine/vehicle. Note that the two sgRNAs are positioned in tandem, 175 and 176 among the ranked sgRNAs. **b**) *DNAJB12*-knockout cells were prepared and used for dose-response assay against gemcitabine. **c**) SURVEYOR assay confirmed indel mutations on the target site of the sgRNA on *DNAJB12* gene. **d**) The monoclonal *DNAJB12*-knockout cell lines were prepared and tested for sensitivity to gemcitabine.

EXCLI Journal 2021;20:1615-1620 – ISSN 1611-2156
Received: October 12, 2021, accepted: December 01, 2021, published: December 06, 2021

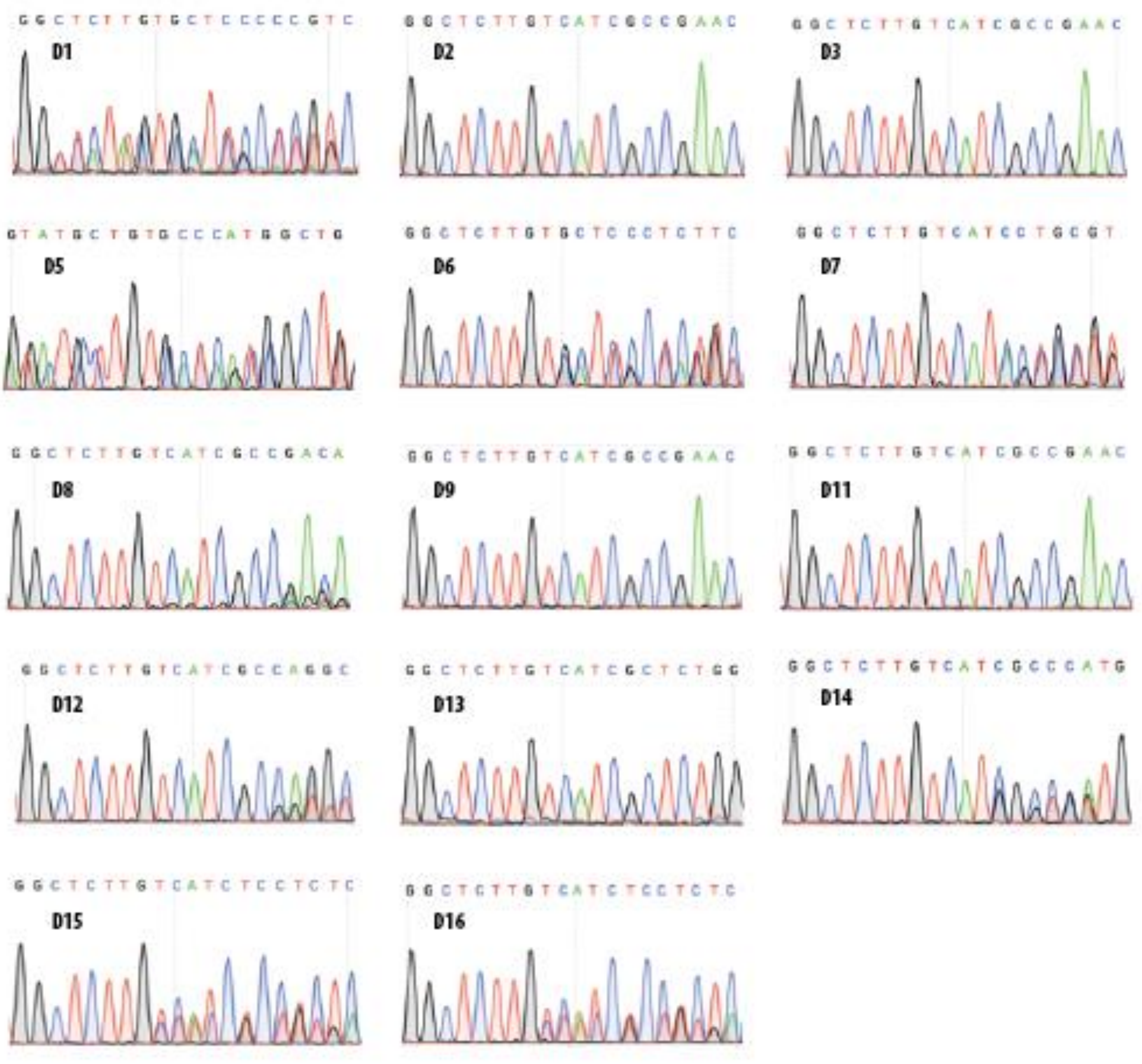

Supplementary Figure 4: The DNAJB12 sgRNA target site. Sanger DNA sequencing chromatogram of the DNAJB12 sgRNA target site on the genome of DNAJB12-knockout cell lines. Note that cell lines D2, D3 and D9, which showed a dramatic increase in sensitivity to gemcitabine compared to the control, have intact target sites.
